# CDC7 is a targetable regulator of advanced prostate cancer

**DOI:** 10.1038/s41598-025-29574-2

**Published:** 2025-12-18

**Authors:** Alifiani B. Hartono, HyeonJi Hwang, Sidharth Paparaju, Shiqin Liu, James D. Brooks, Eva Corey, Tanya Stoyanova

**Affiliations:** 1 Department of Molecular and Medical Pharmacology, University of California Los Angeles, 650 Charles E Young Dr S, Los Angeles, CA 90095 USA; 2 Canary Center at Stanford for Cancer Early Detection, Stanford University, Stanford, CA 94305 USA; 3 Department of Urology, Stanford University, Stanford, CA 94305 USA; 4 Department of Urology, University of Washington, Seattle, WA 98105 USA; 5 Department of Urology, University of California Los Angeles, Los Angeles, CA 90095 USA

**Keywords:** Cancer, Cell biology, Oncology, Urology

## Abstract

Prostate cancer is estimated to contribute to over 35,000 deaths of men residing in the United States, with the majority fatality due to metastatic disease. CDC7 is a kinase that regulates DNA replication and is found elevated during neuroendocrine transdifferentiation in lung and prostate cancer. In this study, we demonstrate that CDC7 is highly expressed in treatment-resistant prostate cancer, with even higher levels observed in treatment-resistant prostate cancer with neuroendocrine phenotype (NEPC). We further identify CDC7 as a critical regulator of prostate tumorigenesis. Downregulation of CDC7 significantly reduces prostate cancer cells growth and invasion *in vitro* and silencing CDC7 suppresses prostate tumor growth *in vivo*. Furthermore, we demonstrate that the inhibition of CDC7 using TAK-931, a selective CDC7 inhibitor, significantly reduces the proliferation, migration, and invasion of aggressive prostate cancer cells. TAK-931 treated prostate cancer cells exhibit an abnormal cell cycle profile, suggesting that CDC7 inhibition induces replication stress and promotes apoptosis. Collectively, our findings demonstrate that CDC7 is a regulator of tumor progression in prostate cancer and represents new therapeutic target in advanced prostate cancer.

**Supplementary Information:**

The online version contains supplementary material available at 10.1038/s41598-025-29574-2.

## Introduction

Prostate cancer is the second leading cause of cancer in men, with more than 300,000 estimated new cases and 35,000 deaths expected in 2025 in the United States^1^. While the 5-year survival rate for localized prostate cancer is relatively high at 97%, those who are diagnosed with metastatic disease are given less than 31% survival rate^2^. Androgen deprivation therapy (ADT) is the current standard of care for metastatic prostate cancer. Although nearly all prostate cancer initially responds to ADT, a subset of these cells adapts and become resistant, leading to the progression of castration resistance prostate cancer (CRPC), a lethal form of the disease. While the predominant histological variant of CRPC is adenocarcinoma (adeno-CRPC), about 20% of CRPCs acquire neuroendocrine phenotype, commonly referred to as neuroendocrine prostate cancer (NEPC)^3–6^. NEPC is highly aggressive and resistant to inhibition of the AR-pathway due to downregulation of AR-expression^6^. Despite current treatment options, metastatic Adeno-CRPC and NEPC are invariably lethal, highlighting the urgent need for identifying new druggable targets and effective treatment modalities.

Changes in pathways that maintain genomic integrity often occur as cancer progresses. Genes that regulate the cell cycle arrest, such as *TP53* and *RB1*, are often altered^7,8^, and high levels of the DNA replication complex proteins, such as members of the minichromosome maintenance complex (MCM) DNA helicase, have been observed in advanced prostate cancer^9^. CDC7 is a conserved serine/threonine kinase that is activated by the regulatory subunits of DBF4^10^. The CDC7 and DFB4 complex (DDK) promotes DNA replication origin firing by site-specific phosphorylation of the MCM2, MCM4, and MCM6 proteins,^11–14^ that induce conformational changes and enable recruitment of the heterotetrameric GINS complex and cell division cycle 45 (CDC45). This protein complex forms the active helicase known as CDC45-MCM2-7-GINS complex (CMG)^15–18^. CDC7 is also a key factor in maintaining genomic stability in response to DNA damage and stalled replication forks^19–22^. Increased CDC7 expression has been observed in many advanced cancer and is associated with poor prognosis in ovarian and triple-negative breast cancer^23^. In prostate cancer model systems, CDC7 promotes the differentiation of adeno-CRPC into NEPC^24^. Newly available CDC7 inhibitors have shown efficacy in various types of cancer and could be effective in treating adeno-CRPC and in preventing the transition to NEPC^25,26^.

In this study, we demonstrated that CDC7 is highly expressed in the majority of advanced prostate cancer cell lines, patient-derived xenografts (PDXs), and adeno-CRPC and NEPC patient samples. Functional studies revealed that depletion of CDC7 impaired the proliferation, migration, and invasive capacity of advanced prostate cancer cells *in vitro* and suppressed tumor growth *in vivo*. These findings suggest the critical role of CDC7 in driving aggressive prostate cancer tumor growth. Inhibition of CDC7 significantly decreased cell proliferation and invasion of prostate cancer cell lines. Taken together, our findings support CDC7 as a promising therapeutic target for adeno-CRPC and NEPC.

## Results

### CDC7 is highly expressed in advanced prostate cancer

**Fig. 1:**
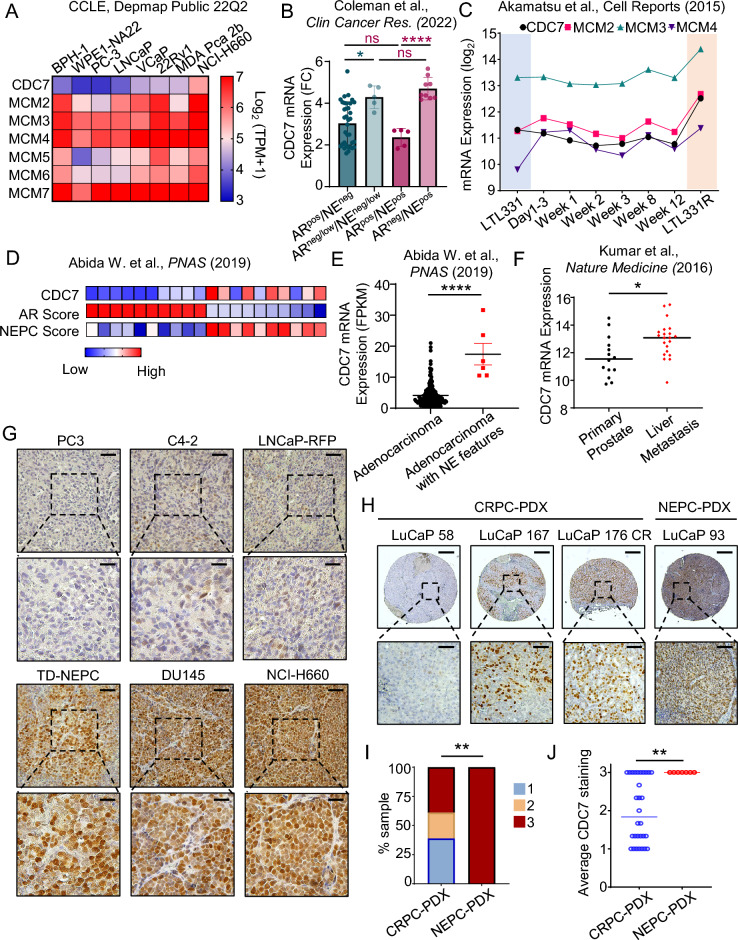
CDC7 is highly expressed in advanced prostate cancer. (**A**) Heatmap of *CDC7* and MCM helicase mRNA levels in benign prostate hyperplasia (BPH-1), a transformed prostate epithelial line WPE1-NA22, and prostate cancer cell lines. ^27^ (**B**) *CDC7* expression in 54 PDX LuCaP models with different metastatic CRPC phenotypes: AR^neg^/NE^neg^ (n = 35), AR^neglow^/NE^neg/low^ (n = 5), AR^pos^/NE^pos^ (n = 5), AR^neg^/NE^pos^ (n = 9)^28^. (**C**) *CDC7* mRNA expression, where LTL331 represents hormonal-naïve prostate adenocarcinoma grafted into NOD/SCID mice and transdifferentiated into LTL331R, which exhibit neuroendocrine features, after host castration^29^. (**D**) Heatmap illustrating the level of *CDC7* mRNA expression and NEPC score from the SU2C/PCF dataset arranged according to the top 10 and bottom 10 patient samples AR score^30^. (**E**) Dot plot depicting *CDC7* mRNA expression according to the pathologic classification from the SU2C/PCF patient dataset as well as (**F**) *CDC7* mRNA expression level in patients’ primary tumors versus metastatic tumors in the liver from Kumar et al. dataset^31^. (*p < 0.05, ****p < 0.0001, mean ± SD, student *t*-test) (**G**) CDC7 protein levels in xenograft tumors derived from prostate cancer cell lines and (**H**, **I**, **J**) LuCaP PDX TMA consisting of CRPC (30 tumors, 90 cores) and NEPC (7 tumors, 21 cores). Scale bars represent 100 µm (top) and 20 µm (bottom) for G, and 250 µm (top) and 50 µm (bottom) for H. (**p < 0.01, z-score test for two population proportions).

To determine the expression level of *CDC7* in prostate cell lines, we utilized the Cancer Cell Line Encyclopedia (CCLE) publicly available dataset^27^. We found that the majority of prostate cancer cell lines express high levels of *CDC7* transcripts when compared to a benign prostate hyperplasia (BPH-1) cell line and a transformed prostate cell line with low malignant potential, WPE1-NA22 (Fig. 1A). The neuroendocrine castration resistant cell line, NCI-H660, had the highest *CDC7* transcript expression as well as higher transcript levels of the MCM helicase proteins. Elevated *CDC7* and MCM helicase expression levels in these cell models, especially the CDC7 direct target MCM2 subunit, correlate with higher proliferative rates observed in clinical prostate cancer with disease progression.

We next assessed the expression of *CDC7* in 54 prostate cancer LuCaP PDX tumors with different metastatic CRPC phenotypes (Fig. 1B)^28^. *CDC7* expression level was higher in PDX tumors with negative or low AR phenotypes compared to PDX tumors with AR positive phenotype. The presence or absence of neuroendocrine (NE) characteristics did not significantly affect *CDC7* expression as PDX tumors with NE high or NE low characteristics have similar *CDC7* expression levels when AR phenotype is the same, suggesting that loss of AR is correlated more with *CDC7* expression than with NE phenotypes. In addition, mRNA analysis of a PDX model of NEPC transdifferentiation revealed increased expression of *CDC7*,*MCM2*, *MCM3*, and *MCM4* as the AR-positive adenocarcinoma model (LTL331) transitioned to the NEPC phenotype (LTL331R) (Fig. 1C)^29^. Similar to the 54 LuCaP PDX models, higher expression of *CDC7* was also correlated with a lower AR-score, higher NEPC score, and neuroendocrine features in patients with metastatic CRPC (Fig. 1D and E)^30^. High expression of *CDC7* mRNA level was also observed in liver metastasis (n = 21) compared to primary prostate tumors (n = 14) taken from a rapid autopsy study (Fig. 1F)^31^. Immunohistochemistry analysis of a panel of prostate cancer xenografts revealed higher CDC7 protein expression in xenografts lacking AR expression and exhibiting a neuroendocrine phenotype, such as the LNCaP Trop2 oncogene driven NEPC model (TD-NEPC)^32^ and NCI-H660, compared to the adenocarcinoma cell lines C4-2 and LNCaP (Fig. 1G). Consistent with the CCLE public mRNA public dataset, PC3 cells did not express high levels of CDC7 even though the cell line moderately expressed the neuroendocrine markers, CgA and NSE, and is also AR-negative^33,34^. DU145, a cell line that does not express AR or neuroendocrine markers, also expressed high level of CDC7 expression. CDC7 protein level expression in these cell lines (Supplementary Fig. 1A) correlated directly with transcript levels that were found in the CCLE public mRNA dataset (Fig. 1A). Furthermore, we evaluated CDC7 protein level in a tissue microarray (TMA) of LuCaP PDX lines comprised of 30 adeno-CRPC and 7 NEPC PDX, with three cores per PDX. Intensity scores were assigned based on staining intensity of the stained cores (Supplementary Fig. 1B). Representative images of intensity scores found in CRPC and NEPC are shown in Fig. 1H. High level expression of CDC7 was observed in all 21 neuroendocrine PDX tumor cores and in 55/90 adeno-CRPC PDX tumor cores (Fig. 1I and J). Collectively, these results demonstrate that high levels of CDC7 expression are associated with the aggressive adeno-CRPC and neuroendocrine prostate cancer phenotypes.

### Downregulation of CDC7 inhibits prostate cancer cell growth and invasion in vitro and in vivo

**Fig. 2:**
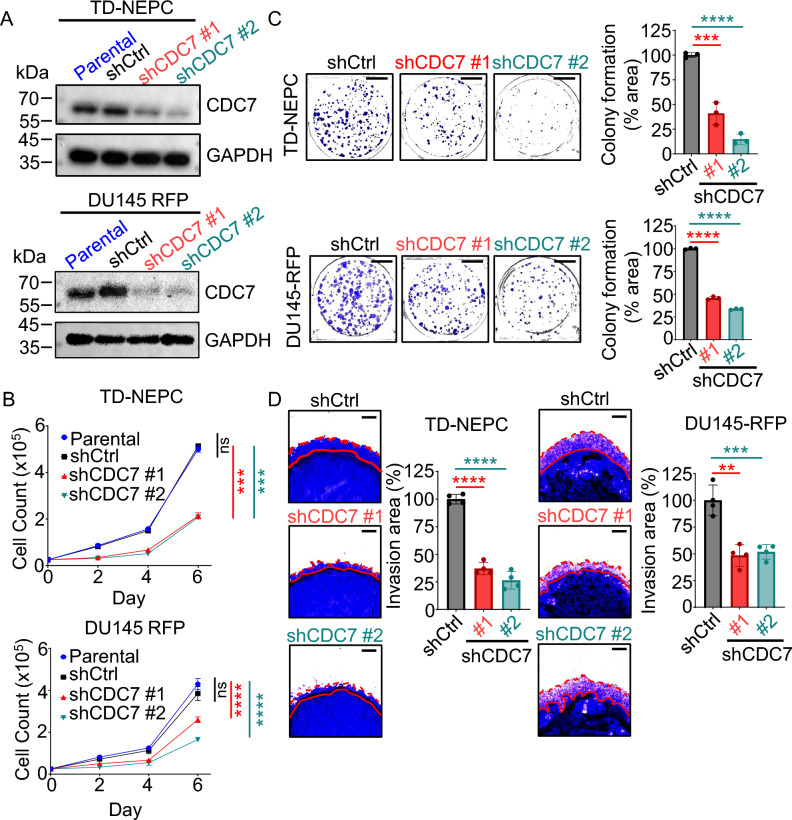
CDC7 knockdown inhibits prostate cancer cells growth and invasion capability *in vitro*. (**A**) CDC7 protein expression in parental, shRNA scrambled (shCtrl) and two unique shRNA constructs targeting CDC7 in TD-NEPC and DU145-RFP cell lines. Protein levels were assessed by Western Blot (cropped images) and GAPDH was used as loading control. (**B**) Growth curve of TD-NEPC parental, shCtrl, shCDC7 #1, shCDC7#2. ns = not significant, ***p < 0.001, ****p < 0.0001, mean ± SD, one-way ANOVA. (**C**) Colony formation assay of TD-NEPC and DU145-RFP shCtrl shCDC7 #1, and shCDC7 #2 cell lines. The percentage of colony area of each well was quantified and normalized to the shCtrl cell lines. (**D**) 3D matrigel drop assay of TD-NEPC and DU145 RFP shCtrl, shCDC7 #1, and shCDC7 #2 cells. The percentage of invaded area was normalized to the shCtrl cells. Scale bar is 500 µm. **p < 0.01, ***p < 0.001, ****p < 0.0001, mean ± SD, student *t*-test.

To determine the oncogenic role of CDC7 in adeno-CRPC and NEPC, CDC7 was depleted in TD-NEPC and DU145 cell lines using two unique shRNAs. Both shRNA constructs reduced CDC7 protein levels compared to an shRNA control and the parental cell line (Fig. 2A). Knockdown of CDC7 decreased the proliferation of TD-NEPC and DU145-RFP cells (Fig. 2B) and mRNA transcripts of proliferation markers, *Ki67* and *PCNA* (Supplementary Fig. 2A). Likewise, depletion of CDC7 reduced colony formation of TD-NEPC and DU145-RFP cell lines by more than 60% compared to shControl and parental cell lines (Fig. 2C).

In addition, CDC7 knockdown decreased the invasion of both cell lines by at least 50% in a 3D Matrigel drop assay, as measured by invasion area (Fig. 2D). The reduced invasion ability of shCDC7 TD-NEPC and DU145 cell lines may be due to the disruption of TGF-β signaling, a well-known promoter of epithelial-to-mesenchymal transition (EMT) in advanced prostate cancer^35^. In both TD-NEPC and DU145 shCDC7 cell lines, the expression of *TGF*-*β1 *were transcriptionally lower than shControl cell lines (Supplementary Fig. 2B). Furthermore, TGF-β1 regulated EMT genes^36^, *SMAD3* and *MMP9*, expressions were also significantly decreased when CDC7 expression is knockdown.

**Fig. 3:**
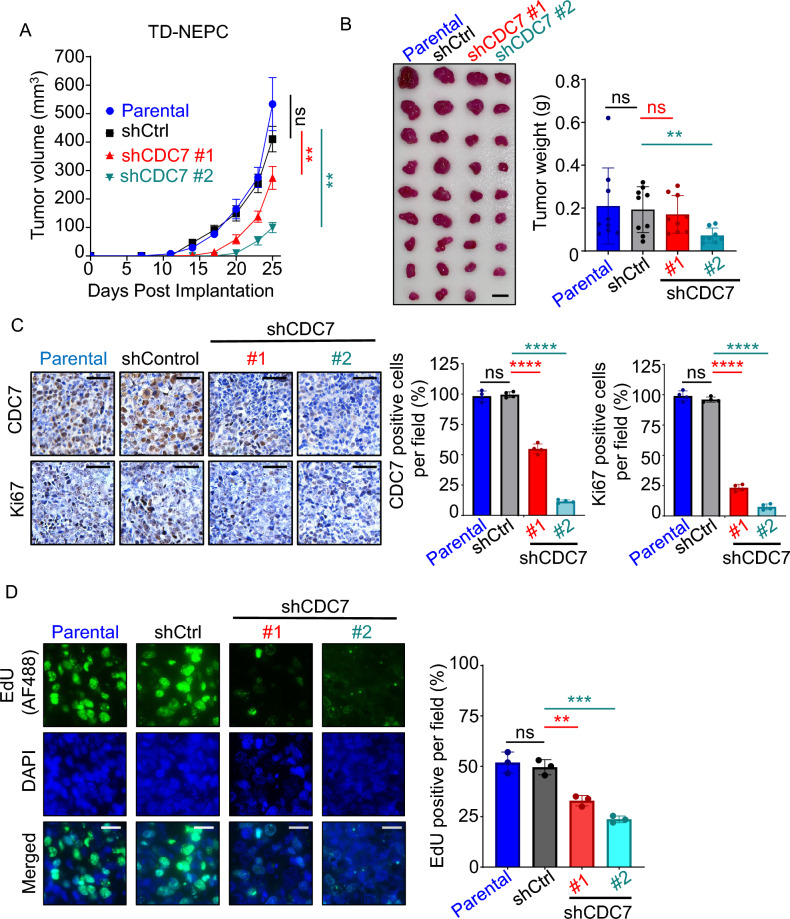
Depletion of CDC7 inhibits growth of prostate cancer xenografts. (**A**) Growth curves of TD-NEPC parental, shCtrl, shCDC7 #1, shCDC7 #2 cell lines implanted subcutaneously in male NSG mice (n = 5/cell lines) based on tumor size [(length x width x height/2)]. At end point, the average size of TD-NEPC parental and shCtrl xenograft tumors were more than 400 mm^3^. (**B**) Collected xenograft tumors and their respective tumor weight **(C)** Immunohistochemical staining of TD-NEPC parental, shCtrl, shCDC7 #1, shCDC7 #2 xenografts for CDC7 and Ki67. Size bar is 25 µm. (**D**) Immunofluorescent images of TD-NEPC parental, shCtrl, shCDC7 #1, shCDC7 #2 xenografts for the presence of EdU. Size bar is 25 µm. ns: not significant, **p < 0.01, ****p < 0.0001, one-way ANOVA for (**A**) and student t-test for (**B**), (**C**) and (**E**).

To determine whether depletion of CDC7 also affects prostate cancer growth *in vivo*, TD-NEPC and DU145-RFP shCDC7 #1 and #2, along with parental control and shControl cell lines, were implanted subcutaneously into the flanks of NOD SCID gamma (NSG) mice. CDC7 knockdown in TD-NEPC and DU145-RFP cell lines was associated with slower xenograft tumor growth and size compared to parental and shControls (Fig. 3A and B, Supplementary Fig. 3A and B). While both parental and shControl xenograft tumors grew more than 400 mm^3^ for TD-NEPC tumors and more than 300 mm^3^ for DU145-RFP tumors, shCDC7 #1 and #2 xenograft tumors were at least half the size of the parental and shControl xenograft tumors. Ki67 expression, a marker of cell proliferation, was also decreased by more than 50% in shCDC7 xenograft tumors (Fig. 3C). Since CDC7 is involved in initiating DNA replication, we utilized EdU, a pyrimidine analog, to measure de novo DNA synthesis in xenograft tumors derived from shCDC7 prostate cancer cell lines. EdU was administered intraperitoneally 8 h prior to euthanizing the mice. Immunofluorescence staining showed significantly decreased EdU-positive cells in both TD-NEPC and DU145-RFP shCDC7-derived tumors compared to parental and shControl tumors (Fig. 3D, Supplementary Fig. 3C). Therefore, CDC7 appears to be important in driving the growth of adeno-CRPC and NEPC and depletion CDC7 expression can hinder proliferation and DNA replication.

### Treatment with a CDC7 inhibitor decreases growth in prostate cancer

**Fig. 4:**
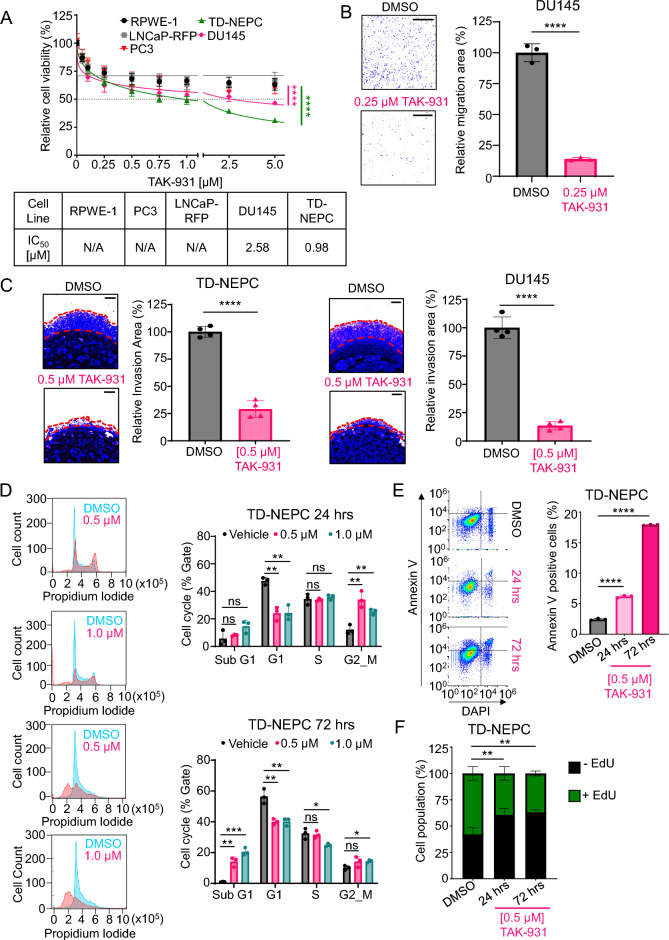
TAK-931 inhibition of CDC7 inhibits growth, migration, and invasion and alters the cell cycle. (**A**) Cell viability of normal prostate epithelial cell line, RWPE-1, and prostate cancer cell lines LNCaP-RFP, PC3, TD-NEPC, and DU145 after 72 h of TAK-931 treatment at various doses. (**B**) DU145 cells treated for 48 h with TAK-931 and transferred to transwell chamber assays to measure migration capability 18 h post plating. (**C**) 3D Matrigel drop assay of TD-NEPC and DU145 after TAK-931 treatment. The percentage of invaded area was normalized to vehicle (DMSO). TD-NEPC cells were treated with TAK-931 for either 24 h or 72 h prior to analysis (**D**) Cell cycle distribution based on Propidium Iodide staining, (**E**) apoptosis and (**F**) DNA replication measured via flow cytometry. Scale bar is 500 µm for (**B**) and (**C**). **p < 0.01 and ****p < 0.0001, mean ± SD, student *t*-test, two-way ANOVA (**A**), Two-tailed z-test (**F**).

To test whether prostate cancer cells are dependent on CDC7 activity for growth and evaluate its potential as a therapeutic target, we evaluated the effects of TAK-931, a CDC7-specific inhibitor, on prostate cancer cell growth^37,38^. Both TD-NEPC and DU145, which express high levels of CDC7, were more sensitive to TAK-931 treatment compared to the adenocarcinoma cell lines including PC3, LNCaP-RFP, and RPWE-1, which express lower CDC7 levels (Fig. 4A). TD-NEPC and DU145 showed significantly lower colony formation ability with TAK-931 treatment compared to vehicle controls (Supplementary Fig. 4A). Inhibition of CDC7 with TAK-931 also significantly reduced cell migration and invasion of DU145 cells compared to vehicle-treated controls (Fig. 4B and Fig. C Right). Likewise, TD-NEPC cells treated with TAK-931 had diminished capacity for invasion in the Matrigel drop assay compared to vehicle treatment (Fig. 4C Left).

We further investigated the effect of CDC7 inhibition by TAK-931 on the cell cycle in TD-NEPC using propidium iodide staining. TD-NEPC cells were treated with either 0.5 µM and 1.0 µM of TAK-931, and samples were collected 24 h and 72 h post treatment. At 24 h, both doses demonstrated accumulation of cells at G2/M phase with a slight increase in Sub-G1 population. By 72 h there was a significant increase in the Sub-G1 population at both doses suggesting induction of apoptosis (Fig. 4D). Indeed, flow cytometry demonstrated significantly increased in Annexin V positive TD-NEPC cells after TAK-931 treatment indicating induction of apoptosis (Fig. 4E).

Treatment of TD-NEPC cells with TAK-931 resulted in fewer EdU-positive cells compared to vehicle indication inhibition of DNA replication (Fig. 4F). Since CDC7 phosphorylates MCM2 at the S40/S41 sites to initiate DNA replication, we confirmed that treatment with TAK-931 resulted in reduced phosphorylation at pMCM2 S40/S41 in both TD-NEPC and DU145 cell lines (Supplementary Fig. 4B), indicating that CDC7 activity is significantly inhibited by TAK-931 in these cells. Collectively, these results demonstrate that Adeno-CRPC and NEPC cell growth is sensitive to inhibition of CDC7 activity and targeting CDC7 activity is a promising therapeutic approach.

## Discussion

Therapeutic strategies for patients with advanced prostate cancer, such as CRPC and NEPC, are only beneficial for a short duration of time due to the development of resistance. Identifying the pathways responsible for resistance is needed to develop additional therapeutic approaches for those patients. Loss of the phosphate and tensin homolog (PTEN) and the retinoblastoma protein (RB1), as well as overexpression of master transcription factors such as the MYC and MYB families, contribute to the increased genetic instability found in hormone-resistant prostate cancer^39–41^. Interestingly, one of the genes that the c-myb transcription factor modulates is CDC7, a regulator of the DNA helicase complex that is required for the initiation of DNA replication and for restarting stalled forks due to replication stress^19–22,42,43^. In previous work, we used proteomics to identify upregulation in DNA replication machinery protein levels, such as the DNA helicase complex MCM2-MCM7 subunits, in an NEPC model^9,32^. Here we demonstrate that *CDC7* mRNA expression level is elevated in both Adeno-CRPC and NEPC cell lines, patient derived xenograft models, and human samples with neuroendocrine features. Furthermore, *CDC7* expression is inversely correlated with AR signaling, making CDC7 an attractive therapeutic target for CRPC and NEPC.

In this study, we depleted CDC7 protein expression in both Adeno-CRPC and NEPC cell line models and demonstrated the dependency of these cells on CDC7 for their growth, their migration and invasion capabilities. Since CDC7 functions in normal cells during cell division, inhibition could be associated with undesirable toxicities. However, CDC7 inhibition in normal cells results in reversible G1 arrest through the p53-dependent cell cycle checkpoint, and cells resume normal division once CDC7 levels and activity are restored^44,45^^,^^46^. Although p53 is often mutated in cancer cells^46^ and is associated with increased CDC7 expression levels^24^, CDC7 is not directly regulated by p53^47^. The neuroendocrine cell line model in this study, TD-NEPC, was derived from LNCaP prostate cancer cell line^32^, which has wild type p53. Despite this, CDC7 inhibition in this cell line produces cell death via apoptosis. Furthermore, in Ewing Sarcoma cell lines, sensitivity to inhibition of CDC7 activity is independent of p53 status^26^. AR loss may drive the change in CDC7 expression more than p53 status since both DU145 and TD-NEPC are AR negative and the status of the AR phenotype of prostate cancer PDX modulate CDC7 expression.

Loss of AR expression or androgen deprivation therapies could also upregulate the expression of TGF-β1 ligand and the expression of TGF-β regulated genes and led to enhanced invasive properties^48–50^. While anti-androgen therapy, such as enzalutamide and bicalutamide, is a standard of care for advanced prostate cancer patients, these anti-androgens could further induce prostate cancer cell invasion by increasing the expression of TGF-β1, SMAD3, and MMP9 in prostate cancer cell lines both *in vitro* and *in vivo*^51^. However, depletion of CDC7 in our advanced prostate cancer cell line led to a decrease in *TGF-β1*, *SMAD3*, and *MMP9* expressions and therefore targeting CDC7 could also be a method to inhibit TGF-β driven metastasis caused by ADT.

TAK-931 is a CDC7-selective kinase inhibitor that has anti-proliferative activity across various cancer cell lines and causes tumor growth inhibition in multiple cancer xenograft models, including human colorectal, lung, ovarian, and pancreatic cancer^37^. In a recent clinical trial (NCT02699749), TAK-931 was shown to decrease pMCM2 levels in skin and tumor samples in a dose-dependent manner, demonstrating on-target efficacy^52^. Our data demonstrate that TAK-931 could have a role in treating advanced prostate cancer, particularly adeno-CRPC and NEPC models that exhibit high expression of CDC7. Inhibition of CDC7 in prostate cancer cell lines and xenograft models led to cell cycle arrest at G2/M, induction of apoptosis, and inhibition of migration and invasion. Together, our data suggest that targeting CDC7 activity could be a therapeutic strategy in adeno-CRPC and NEPC.

In summary, our study demonstrated that CDC7 is overexpressed in advanced prostate cancer, and that depletion of CDC7 caused significant impairment in cell growth *in vitro* and *in vivo*. Adeno-CRPC and NEPC models with elevated levels of CDC7 expression rely on CDC7 activity to maintain growth and survival, and inhibition of CDC7 activity with TAK-931 caused cell cycle arrest and induces apoptosis. Therefore, targeting CDC7 activity in adeno-CRPC and NEPC patients could be a promising therapeutic approach.

## Methods

### Datasets

Raw data from all public datasets were downloaded from cBioPortal for Cancer Genomics^53^. mRNA levels of CDC7 and MCM2-7 from prostate cancer cell lines were taken from the Cancer Cell Line Encyclopedia^27^. CDC7 mRNA level from 54 LuCaP PDX lines was obtained from Coleman et al. dataset and mRNA expression of MCM2/3/4 and CDC7 were acquired from Akamatsu et al. study^29,54^. CDC7 mRNA expression, as well AR score, NE score, and pathology classification, were downloaded from the Abida et. al SU2C/PCF dataset^30^. CDC7 mRNA expression from primary prostate and metastatic prostate cancer sites were obtained from Kumar et al.^31^.

### Cell lines and cell culture

LNCaP (ATCC-CRL-1740, RRID: CVCL_0395), DU145 (ATCC-HTB-81, RRID: CVCL_0105), PC-3 (ATCC-CRL-1435, RRID: CVCL_0035), and RWPE-1 (ATCC-CRL-11609, RRID:CVCL_3791) were purchased from American Type Culture Collection (ATCC). TD-NEPC (Trop2-driven NEPC) and LNCaP-RFP cell lines were previously established by infecting LNCaP parental cells with a Trop2-overexpression lentiviral vector containing red-fluorescent protein (RFP) or with RFP alone^32^. TD-NEPC, LNCaP-RFP, DU145, and PC3 cells were maintained in RPMI medium supplemented with 10% FBS, 100 U/mL penicillin, 100 µg/mL streptomycin, and 1X glutamax (Thermofisher). Cell lines were previously authenticated by Stanford Functional Genomics Facility based on Short Tandem Repeat (STR) profiling and routinely checked for mycoplasma contamination by PCR-based assay^55^. TAK-931 was purchased from MedChemExpress (cat #HY-100888).

### Generation of CDC7-KD cells

Viral particles were generated by transfecting lentiviral vectors and viral envelopes and packaging vectors into 293 T cells via calcium phosphate. TD-NEPC and DU145-RFP cell lines were transduced with polybrene (10 µg/mL) and generated virus particles. Transduced cell lines were selected with 1 µg/mL puromycin for 9 days and knockdown efficiency was confirmed through western blot. Two independent shRNA pLKO.1 lentiviral vectors against CDC7 were purchased from Millipore Sigma (TRCN0000350364, target sequence: TTCAAGAAGTACGGGAATATA and TRCN0000003171, target sequence: CCTAATCTGTTTGGTAAGTAT), and pLKO.1 control scramble was a gift from David Sabatini (Addgene plasmid # 1864; http://n2t.net/addgene:1864 ; RRID:Addgene_1864).

### Cell proliferation and viability assay

For cell proliferation, 2.5 × 10^4^ of TD-NEPC (parental, shControl, shCDC7 #1, and shCDC7) and DU145-RFP (parental, shControl, shCDC7 #1, and shCDC7 #2) cells were seeded in 6-wells plate in triplicate for each time point (Day 2, 4, 6). At each time point, cells were trypsinized and viable cells were stained with trypan blue and counted with a hemocytometer. For cell viability assays, 5 × 10^3^ (TD-NECP, LNCaP-RFP, PC3 and RPWE-1) and 2.5 × 10^3^ (DU145) cells were seeded in 96-well plates. After 24 h, cells were treated with serial dilutions of TAK-931 (from 5 to 0.06125 μM) and cell viability was quantified using Cell-Titer Blue (Promega).

### RNA-extraction and real-time qPCR assays

Total RNAs were extracted with TRIzol Reagent (Invitrogen, Carlsbad, CA), and 1 µg total RNAs were reverse transcribed into first-strand cDNA (iScript cDNA synthesis kit, Bio-Rad, Hercules, CA). Relative transcript levels were analyzed by real-time PCR using SYBR Green (SsoAdvanced Universal SYBR Green Supermix, Bio-Rad) and calculated by the comparative cycling threshold (Ct) method normalized against human β-actin. The following were the primers used in this study: β-actin (human) (F: 5’ CATGTACGTTGCTATCCAGGC 3’; R: 5’ CTCCTTAATGTCACGCACGAT 3’); CDC7 (human) (F: 5’ GGAAAACTGCCAGTTCTTGCCC 3’; R: 5’ GGCACTTTGTCAAGACCTCTGG 3’); MKI67 (human) (F: 5’ GAAAGAGTGGCAACCTGCCTTC 3’; R: 5’ GCACCAAGTTTTACTACATCTGCC 3’); PCNA (human) ( F: 5’ CAAGTAATGTCGATAAAGAGGAGG 3’; R: 5’ GTGTCACCGTTGAAGAGAGTGG 3’); TGF-β1 (human) (F: 5’ TACAGCACGGTATGCAAGCC 3’; R: 5’ GCAACCGATCTAGCTCACAGAG 3’); SMAD3 (human) (F: 5’ TGAGGCTGTCTACCAGTTGACC 3’; R: 5’ GTGAGGACCTTGTCAAGCCACT 3’); MMP9 (human) (F: 5’ GCCACTACTGTGCCTTTGAGTC 3’; R: 5’ CCCTCAGAGAATCGCCAGTACT 3’).

### 3D Matrigel drop invasion assay

For all cell lines, 5 × 10^4^ cells were resuspended in 10µL of Matrigel (Corning, cat# 354,234) per drop and plated on 24-wells plate as previously described^56^, with 4 wells for each condition or cell line. Media was changed every 72 h with the appropriate condition. At day 6, the drops were fixed with methanol and stained with 0.01% crystal violet before imaging. Invasion areas were quantified by measuring the cell migration area outside of the drop, and invasion capability of cancer cells was normalized to the controls.

### Migration chamber assay

DU145 cells were pre-treated with vehicle (DMSO) or 1.25 µM of TAK-931 for 48 h before seeding 2 × 10^5^ of cells to the upper migration chamber with serum free media and vehicle or 1.25 µM of TAK-931. The lower chamber was filled with 700 µL of media containing 10% FBS and vehicle or 1.25 µM of TAK-931. Three chambers were used per condition. Cells were allowed to migrate for 18 h, before they were fixed in methanol and stained with 0.01% crystal violet for 20 min. Chambers were washed with water twice and air dried overnight. 3 fields were imaged per chamber and Image J (RRID: SCR_003070) was used to quantify the migration percentage area.

### Western Blot

Cells were lysed in RIPA lysis buffer supplemented with protease and phosphatase inhibitor (Thermofisher) and total protein lysates were quantified using the BCA assay. Protein lysates were mixed with sample buffer prior to boiling at 95 °C for 10 min. 15ug of total protein lysates were run on a gradient of 8–16% SDS-PAGE gel and transferred onto a nitrocellulose membrane. The membrane was blocked in 5% w/v non-fat milk, or 5% w/v bovine serum albumin (BSA) for phosphorylated primary antibodies, in TBS-T for one hour prior to overnight incubation with the following primary antibodies: anti-GADPH (1:1000 dilution, Santa Cruz Biotechnology Cat# sc-47724, RRID:AB_627678), anti-CDC7 (1:1000 dilution, Santa Cruz Biotechnology Cat# sc-56275, RRID:AB_831150), and anti-MCM2 (1:1000 dilution,(Santa Cruz Biotechnology Cat# sc-73572, RRID:AB_1126071), and anti-pMCM2 S40/S41 (1:1000 dilution, Bethyl Cat# A300-788A, RRID:AB_577207). Secondary mouse-HRP conjugated antibody (PI31432, 1:5000 dilution) and secondary rabbit-HRP conjugated antibody (PI31462, 1:5000 dilution) were purchased from Fisher Scientific. Western Blots were developed with Pierce ECL Western Blotting Substrate (Thermofisher) and imaged using Bio-Rad Chemidoc XRS Gel Imaging System (RRID:SCR_019690) and analyzed using Image Lab Software (RRID:SCR_014210).

### Immunohistochemical and immunofluorescence staining

Formalin-fixed paraffin-embedded (FFPE) tissues were deparaffinized at 65 °C for 1 h. After rehydration, antigen retrieval was performed by heating the sections to 95 °C for 20 min in 10 mM sodium citrate (pH 6.0). For immunohistochemical staining, endogenous peroxidase activity was blocked in 3% hydrogen peroxide for 5 min. The sections were blocked in 2.5% horse serum in 1xPBS. Sections were incubated overnight at 4 °C with the indicated primary antibodies. Secondary antibodies were purchased from Vector Labs (MP-7452 and MP-7401) and sections were incubated with the appropriate secondary Streptavidin-HRP (Vector Labs; SA-5004; 1:200 dilution) and visualized with DAB substrate (Dako). The sections were counterstained with hematoxylin, dehydrated, and mounted with cover slips. The primary antibodies used were: anti-CDC7 (1:100 dilution, Santa Cruz Biotechnology Cat# sc-56275, RRID:AB_831150) and anti-Ki67 (1:100 dilution(Santa Cruz Biotechnology Cat# sc-23900, RRID:AB_627859). For EdU immunofluorescence staining, after the antigen retrieval step, sections were incubated with Click-It Reaction mix [100 mM Tris–HCl pH 7.6, 4 mM CuSO4, 2.5 µM AF-488 Azide (S1830, Lumiprobe)], and 100 mM sodium ascorbate) for 30 min at room temperature in the dark. Sections were washed with PBS twice and then incubated with DAPI solution (300 nM DAPI in PBS) for three minutes at room temperature in the dark. Sections were washed with PBS three times before mounting. Slides were imaged at 20X magnification (Leica) and three images were taken per slide and used to quantify EdU-positive cells.

#### Cell cycle analysis with propidium iodide and EdU

2 × 10^5^ TD-NEPC cells were seeded in each well of 6 well plates for the 24 h timepoint and 5 × 10^5^ TD-NEPC cells were seeded in 60cm^3^ plates for the 72 h timepoint. Three wells in 6-wells plate or three 60cm3 plates were used for each condition (vehicle (DMSO),0.5 µM, 1 µM TAK-931). At least three biological replicates were done for each experiment. After treatment, cells were collected and washed twice with PBS and fixed with 70% cold ethanol at 4 °C overnight. The next day, cells were pelleted and washed twice with PBS and resuspended in 0.5 mL propidium iodide staining solution (10 µg/mL propidium iodide and 10ug/mL RNase A in PBS) at 4 °C for 10 min protected from light. Cells were analyzed by Thermo Fisher Attune Nxt Flow Cytometer (RRID:SCR_019590) and data was analyzed using FlowJo (RRID:SCR_008520). To quantify DNA replication, cells were pulsed with 20uM of EdU (A10044, Thermofisher) for 1 h prior to collection and fixed overnight at 4 °C with 70% cold ethanol. Fixed cells were then washed twice with PBS and incubated with AF-488 Click-It Reaction mix [100 mM Tris–HCl pH 7.6, 4 mM CuSO4, 2.5 µM AF-488 Azide (S1830, Lumiprobe)], and 100 mM sodium ascorbate) for 30 min at room temperature in the dark. Cells were washed twice with PBS before staining with propidium iodide and analyzed using flow cytometry as described previously.

#### Annexin V

To quantify apoptotic cells, TD-NEPC cells were seeded and treated as described above. At 24 h and 72 h, adherent cells and detached cells in the media were collected and washed once with PBS before resuspending in 100µL Annexin V binding buffer (V13246, Invitrogen) and adding 5 µL of Annexin V-AF647 (A23204, Invitrogen). Samples were incubated at room temperature for 15 min in the dark. Cells were washed twice with binding buffer and resuspend in 400 µL of binding buffer containing DAPI (40 ng/mL). Cells were analyzed with flow cytometry as described previously.

#### Colony formation assay

TD-NEPC and DU145 (shControl, shCDC7 #1, shCDC7 #2) cells (200 cells per well) were seeded in 12-well plates and the media was changed every 3 days. After 9 days, the cells were fixed with methanol and stained with 0.01% crystal violet. Image J was used to quantify the percentage of area occupied by the colonies in each group, and measurements were normalized to shControl.

## Supplementary Information

Supplementary Information.

## Data Availability

The data analyzed in this study were obtained from Gene Expression Omnibus (GEO) at GSE199596 and cBioPortal for Cancer Genomics.

## Declarations

### Animals and in vivo tumor growth

For tumor implantation, cancer cells (1 × 10^6^) were resuspended in 50 µL of PBS and mixed with 50 µl of 100% Matrigel and implanted subcutaneously in both flanks of male NOD/SCID/IL-2Rγnull (NSG) mice. The tumor length (L), width (W), and height (H) were measured every three days, and tumor volumes were calculated (½ LxWxH). At end point, mice were euthanized by administering CO2 in a compressed commercial cylinder with a flow meter rate of 30–70% of the chamber volume/minute for at least 5 min. A cervical dislocation was used as a secondary euthanasia method. Tumors were collected, weighed, and processed for downstream analyses by overnight fixation with 10% buffered formalin followed by tissue processing and embedding in paraffin (FFPE).
