# Supplementary material for: CDC7 is a targetable regulator of advanced prostate cancer
Source: Sci Rep. 2025 Dec 18;16:308. doi: 10.1038/s41598-025-29574-2 (PMC12770614; doi:10.1038/s41598-025-29574-2)
Supplement: Supplementary file 1 — Supplementary Information. [file 41598_2025_29574_MOESM1_ESM.pdf]

Supplemental Figure 1

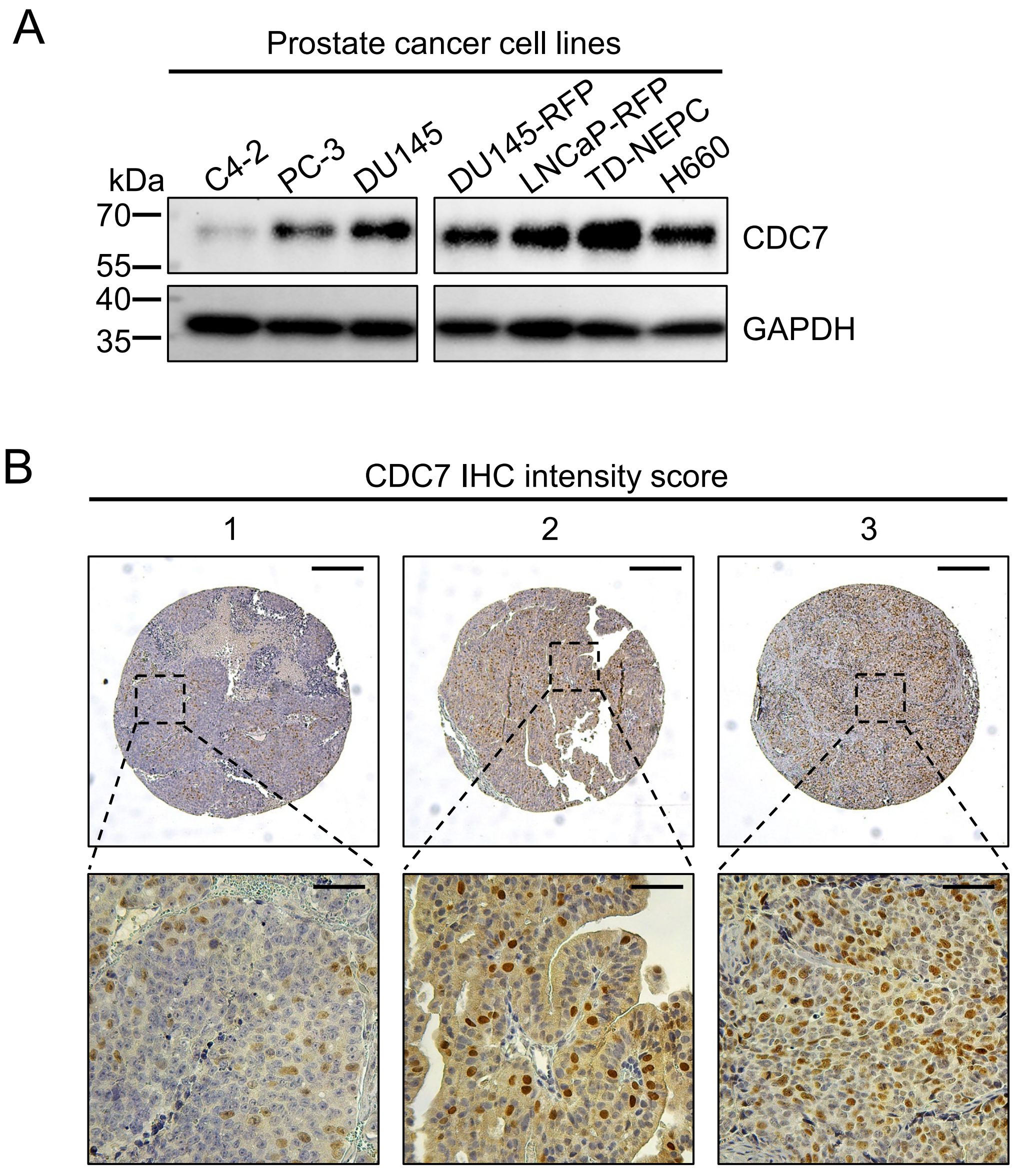

Supplemental Figure 1. **(A)** CDC7 protein level expression across prostate cancer cell lines. **(B)** Representative score of TMA cores. Size bar is 250 μm (top panel) and 50 μm (bottom panel).

Supplemental Figure 2

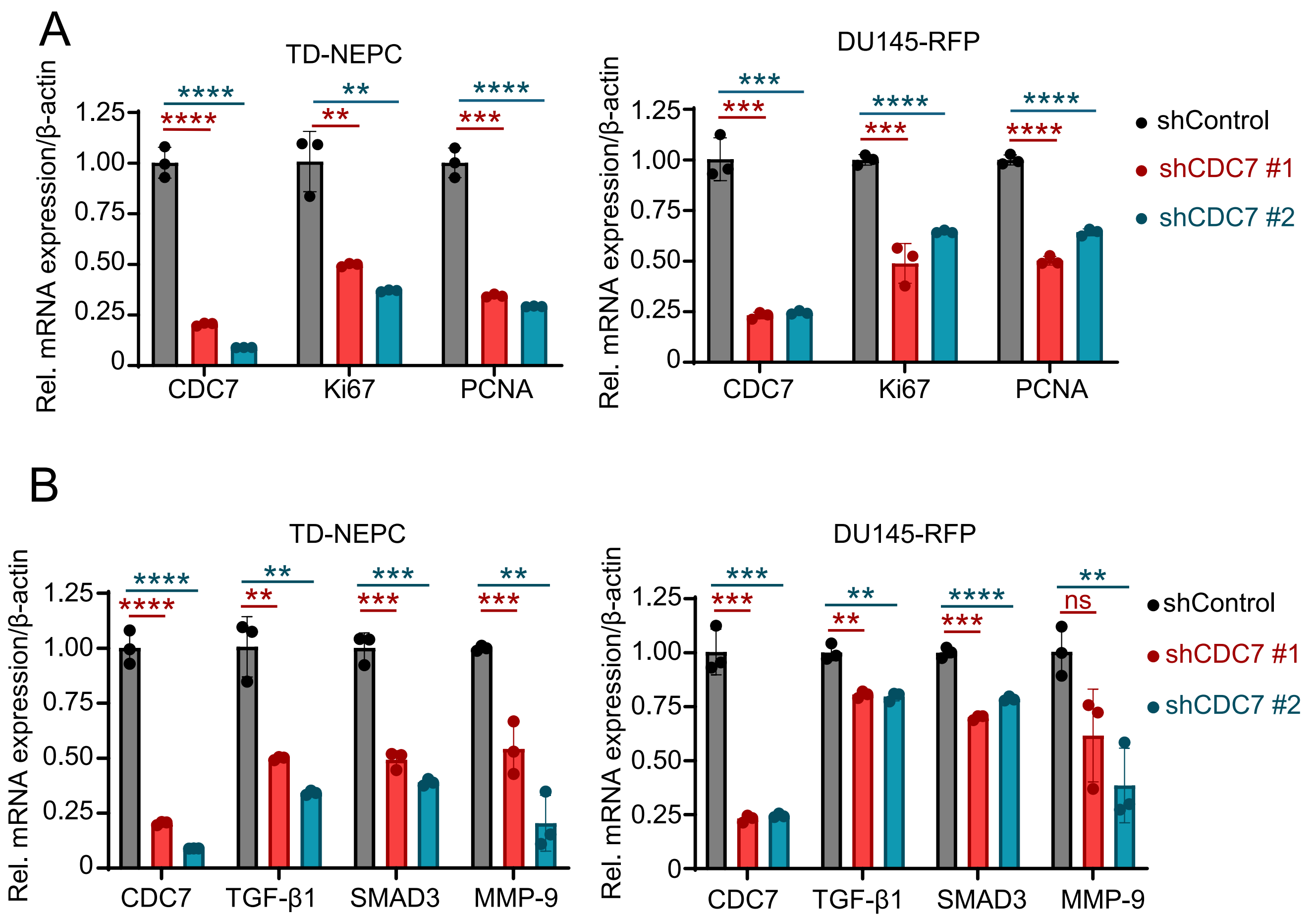

Supplemental Figure 2. (A) Relative mRNA expressions of CDC7, Ki67, PCNA and **(B)** TGF-β1, SMAD3, and MMP-9 in TD-NEPC and DU145-RFP shControl, shCDC7 #1, and shCDC7 #2 cell lines. mRNA expression in shCDC7 #1 or #2 were compared to their respective shControl cell line. ns: not significant, $^{**}p<0.01$, $^{***}p<0.001$, $^{****}p<0.0001$, mean ± SD, student *t*-test.

Supplemental Figure 3

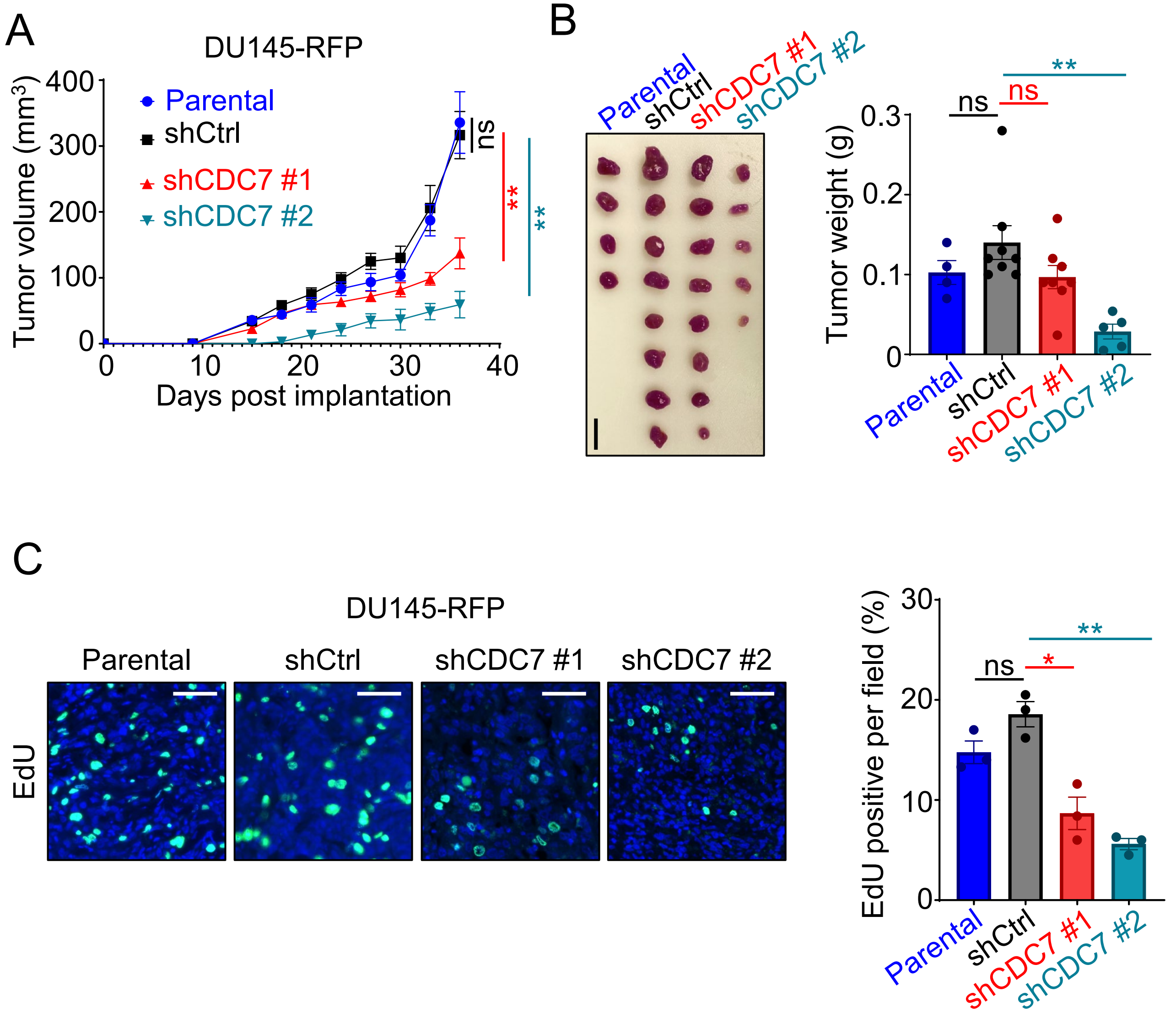

Supplemental Figure 3. (A) DU145-RFP parental, shCtrl, shCDC7 #1, shCDC7 #2 cell lines were subcutaneously implanted in male NSG mice (n=5/cell lines) and growth of tumors were calculated every three days [(length x width x height/2)]. **(B)** Collected xenograft tumors and their respective tumor weight. Size bar: 1 cm **(C)** Immunofluorescent images of TD-NEPC parental, shCtrl, shCDC7 #1, shCDC7 #2 xenografts for the presence of EdU. Size bar is 40 µm. ns: not significant, *p<0.05, **p<0.01, mean ± SEM, one-way ANOVA for (A) and student *t*-test for (B) and (C).

Supplemental Figure 4

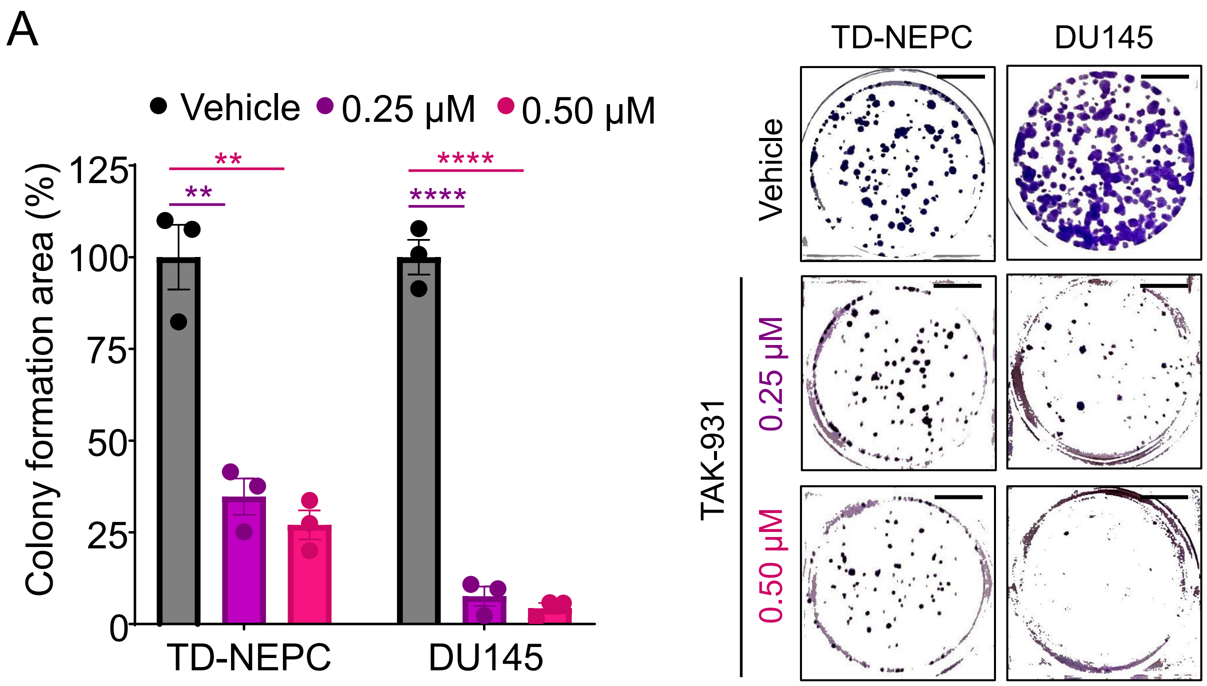

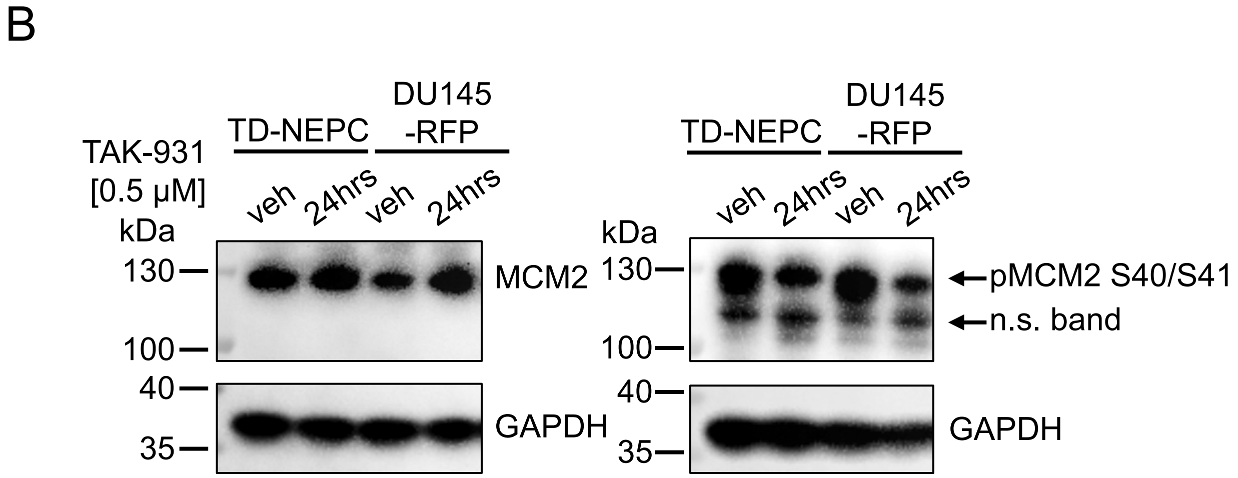

Supplemental Figure 4. (A) Colony formation assay of TD-NEPC and DU145 cell lines treated with 0.25 μM or 0.5 μM TAK-931, or vehicle (DMSO). The percentage of colony area of each well was quantified and normalized to the vehicle treatment group. Size bar: 1 cm **(B)** Western blot of TD-NEPC, DU145, and DU-145 Trop2OV cell lines after 24 hours treatment with 0.5 μM TAK-931 or vehicle (DMSO) for the protein expression of total MCM2 and pMCM2 S40/S41.**p<0.01, ***p<0.001, ****p<0.0001, mean ± SEM, student *t*-test.

Figure 2A uncropped western blot

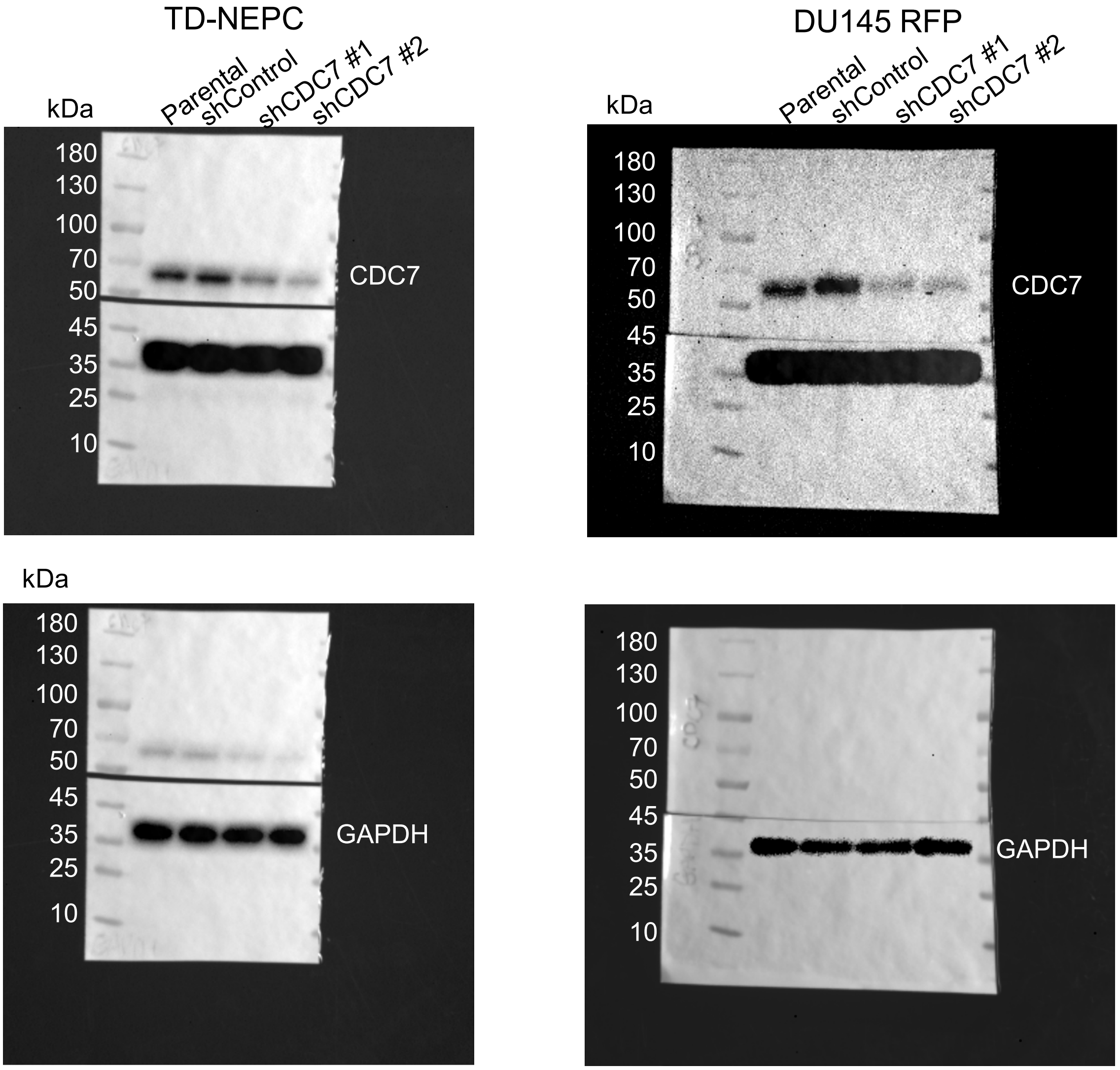

Note: Same developed picture for both CDC7 and GAPDH. CDC7 at longer exposure; GAPDH at shorter exposure.

Supplemental Figure 1A uncropped western blot

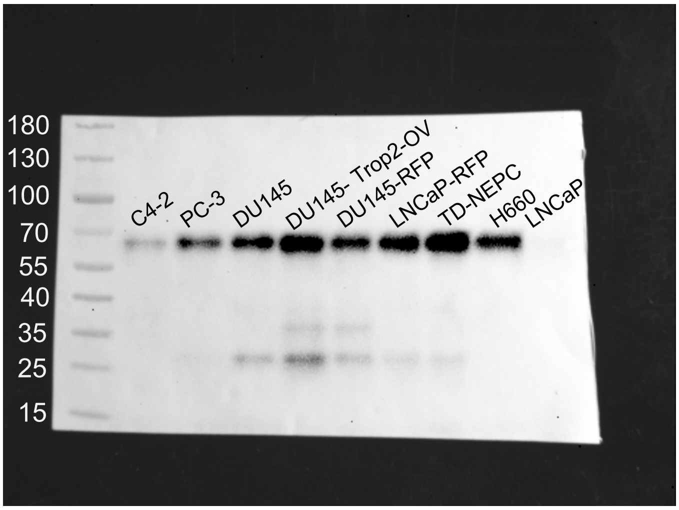

CDC7

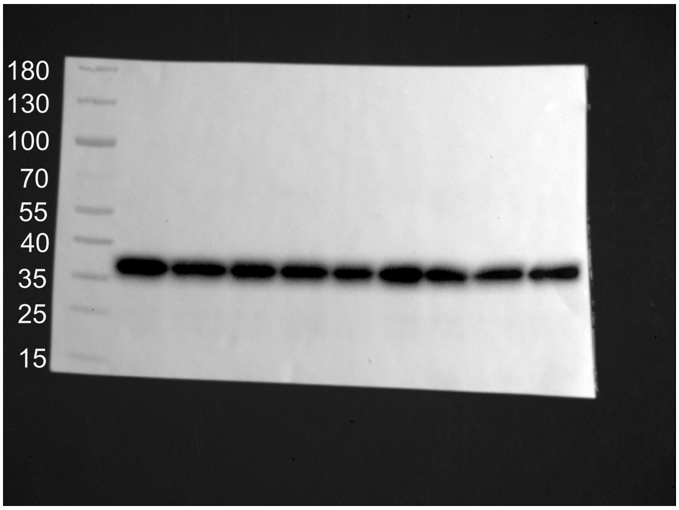

GAPDH

Western blot membrane was first probe for CDC7, imaged, and then stripped and incubated with GAPDH, and then imaged.

Supplementary Figure 4B uncropped western blot

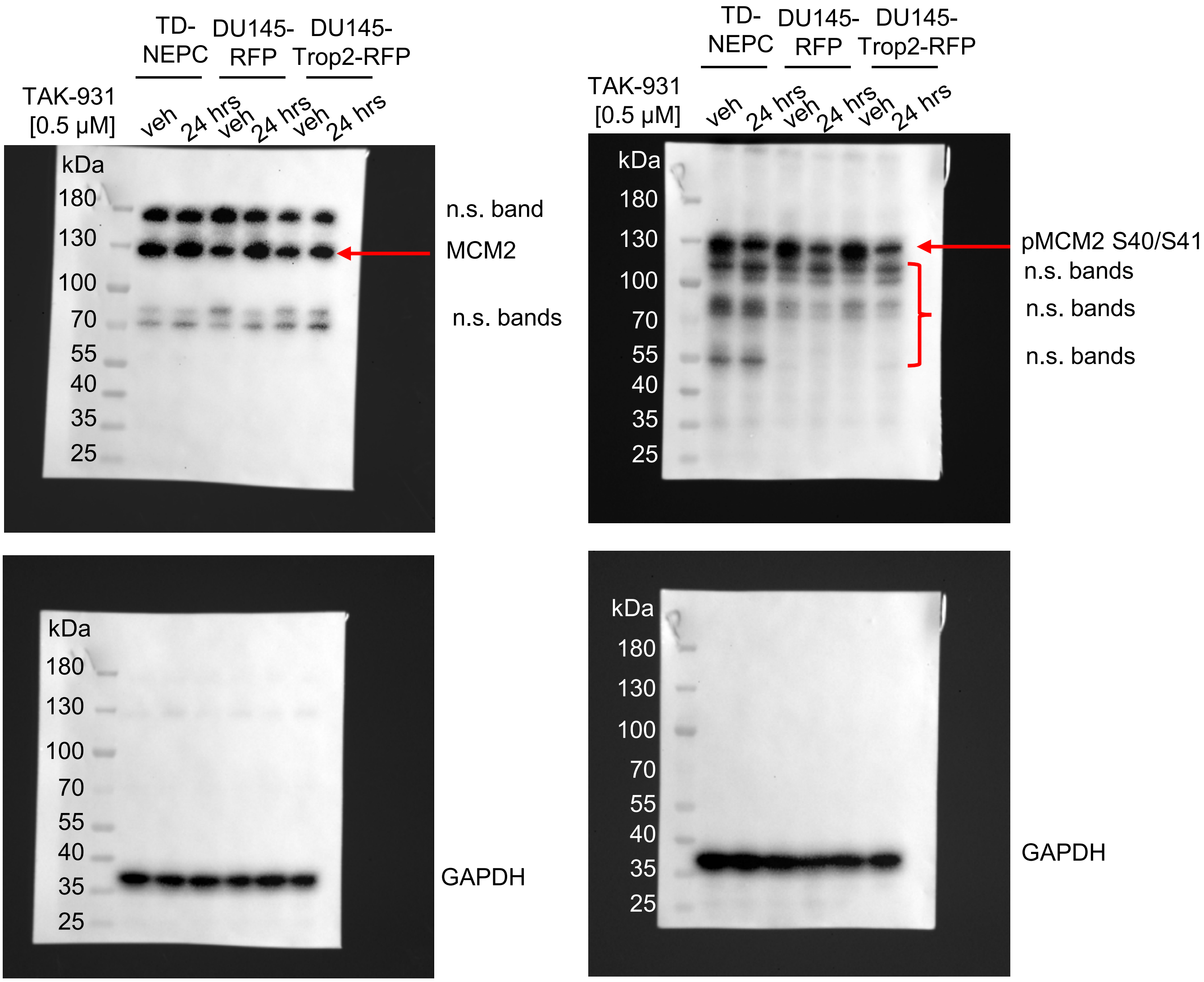

Note: Same western blot membrane– first incubated with MCM2 or pMCM2 S40/S41, imaged, then stripped, and incubated with GAPDH, and imaged
